# The regulation of hematopoietic stem cell populations

**DOI:** 10.12688/f1000research.8532.1

**Published:** 2016-06-28

**Authors:** Hector Mayani

**Affiliations:** 1 Hematopoietic Stem Cells Laboratory, Oncology Research Unit, IMSS National Medical Center, Mexico City, Mexico

**Keywords:** hematopoietic progenitor cells, precursor cells, hematopoietic stem cell niche

## Abstract

Evidence presented over the last few years indicates that the hematopoietic stem cell (HSC) compartment comprises not just one but a number of different cell populations. Based on HSCs’ proliferation and engraftment potential, it has been suggested that there are two classes of HSC, with long- and short-term engraftment potential. HSC heterogeneity seems to involve differentiation capacities as well, since it has been shown that some HSC clones are able to give rise to both myeloid and lymphoid progeny, whereas others are lymphoid deficient. It has been recognized that HSC function depends on intrinsic cell regulators, which are modulated by external signals. Among the former, we can include transcription factors and non-coding RNAs as well as epigenetic modifiers. Among the latter, cytokines and extracellular matrix molecules have been implicated. Understanding the elements and mechanisms that regulate HSC populations is of significant relevance both in biological and in clinical terms, and research in this area still has to face several complex and exciting challenges.

## Introduction

The term hematopoietic stem cell (HSC) refers to an immature cell, residing in the bone marrow, which is capable of both self-renewal and differentiation into all of the different blood cell types. Evidence presented over the last few years, however, indicates that the HSC compartment comprises not just one but a number of different cell populations. This, of course, has both biological and clinical implications. Accordingly, there is great interest in elucidating the identity of each of these cell populations and defining their biological differences and similarities in molecular, immunophenotypic, and functional terms.

## HSCs: basic principles

Although the concept of a primitive, immature cell common to all of the different blood cell lineages (erythrocytes, leucocytes, and platelets) was presented in the first decades of the 20 ^th^ century, it wasn’t until work by James Till and Ernest McCulloch in the early 1960s that the existence of such a stem cell was demonstrated ^1, 2^. The work by Till and McCulloch, in Toronto, Canada, together with that of Metcalf and colleagues, a few years later in Melbourne, Australia, showed that the hematopoietic system could be subdivided into four separate compartments: HSCs (comprising the most immature cells, those capable of self-renewal), hematopoietic progenitor cells (HPCs; those unable to self-renew, but with a large proliferative potential and multilineage, bilineage, or monolineage differentiation capacities), precursor cells (those immature cells that can already be identified through their morphology), and mature blood cells (those present in circulation) ^3^.

Although most of what we know about HSC biology comes from studies in animal models, mostly in mice, it has become evident that human HSCs follow similar biological patterns ^4, 5^. HSCs cannot be identified by morphological criteria; instead, their identification is based on both immunophenotypic analysis and functional assays ^6^. Murine HSCs express antigens such as Sca-1, CD117, and CD150 and do not express CD48; human HSCs, on the other hand, express CD34, CD49f, CD90, and CD117 and do not express CD38 ^4, 7^. In both cases, HSCs do not express any lineage-restricted antigen, so they are referred to as lineage-negative (Lin ^-^) cells ^4, 7^. It is noteworthy that within the human HSC pool a CD34-negative population has also been identified (CD34 ^-^ CD38 ^-^ Lin ^-^), whose cycling status (dormancy) suggests that it is located at the apex of the HSC compartment ^8, 9^. Apart from the expression of specific cell surface markers, HSCs can also be identified by their ability to efflux certain fluorescent dyes, such as Rhodamine-123 (Rho) and Hoechst 33342; thus, they are known as Rho ^-/low^ cells or side population (SP) cells ^7^. The latter form a characteristic cluster of events located off to the lower left side in dual wavelength fluorescence-activated cell sorting (FACS) dot-plot profiles.

Assays to determine the number and functional integrity of HSCs include both *in vivo* and *in vitro* systems. The former consist of introducing HSCs into irradiated animals and determining the ability of such cells to repopulate the hematopoietic system of the host after several weeks post-transplant. This approach is based on the experiments described by Till and McCulloch ^1^, although refined modifications have been introduced into the experimental system during the last few decades ^10^. When using human HSCs, the recipient must be an immunodeficient animal (for instance, severe combined immunodeficient [SCID], non-obese diabetic [NOD]-SCID, or NOD SCID Gamma [NSG] mice), so there will not be rejection mediated by the immune cells of the host ^11^. *In vitro* systems, on the other hand, are based on the ability of HSCs to initiate and sustain hematopoietic cell production for several weeks in cultures containing a stromal cell layer in the presence or in the absence of exogenous cytokines ^12, 13^. It is worth noting, however, that this latter method does not necessarily prove that the cells sustaining hematopoiesis *in vitro* are actual HSCs; thus, to date, the *in vivo* repopulation assay is the only method validated to detect and measure actual HSC function.

It is of particular importance to mention that recent work from Camargo’s and Rodewald’s groups has presented evidence indicating that during steady-state conditions, hematopoiesis is sustained by thousands of long-lived progenitors, rather than by actual HSCs ^14, 15^. These findings suggest that HSC function may not be as critical as previously thought for unperturbed hematopoiesis; in contrast, HSC activity seems to be of great relevance during stress hematopoiesis (e.g., post-hematopoietic cell transplants).

## One or several HSC populations?

Since its conception, the idea that the HSC compartment comprises a homogeneous cell population prevailed for some time; however, studies in animal (murine) models, reported over the last several years, demonstrated that some of the cells contained within the HSC pool are responsible for long-term engraftment, whereas others induce a transient, short-term engraftment ^4, 6, 16^. Some groups have even suggested that, based on their engraftment potential, there are three classes of HSC: those with long-, intermediate, and short-term engraftment potential ^17^. Thus, it is now recognized that the HSC population is heterogeneous, comprising several HSC subsets differing in their repopulation capacities and cycling properties ^18– 20^.

As expected, similar HSC populations also seem to exist in humans. Indeed, work from John Dick’s laboratory in Toronto has shown that human HSCs capable of long-term engraftment express CD34, CD49f, and CD90 and, of course, lack the expression of CD38 and any lineage-restricted antigen; thus, they are defined as CD34 ^+^ CD45RA ^-^ CD49f ^+^ CD90 ^+^ CD38 ^-^ Lin ^-^ cells (LT-HSCs). Loss of expression of CD49f and CD90 gives rise to transiently engrafting multipotent progenitors (MPPs [CD34 ^+^ CD45RA ^-^ CD49f ^-^ CD90 ^-^ CD38 ^-^ Lin ^-^ cells]) ^21^. Based on the genomic analysis performed by the same group on these populations, 70 genes were found to be differentially expressed between HSC subsets and MPPs, whereas 500–3000 genes were differentially expressed when comparing HSCs and more mature, committed progenitors ^21^. All these findings clearly indicate the existence of not one but several populations within the HSC compartment.

HSC heterogeneity seems to involve not only proliferative potentials, as mentioned above, but differentiation capacities as well. Work by different groups, including those of Muller-Sieburg and colleagues, Eaves and colleagues, and Suda and colleagues, has demonstrated that among murine HSCs, some clones are biased towards the production of myeloid cells (α-HSCs), whereas some are biased towards the production of lymphoid cells (γ/δ-HSCs); furthermore, others show a balanced capacity towards the production of both myeloid and lymphoid cells (β-HSCs) ^17, 21– 24^. It is noteworthy that the relative proportion of each one of these HSC subsets varies throughout development. For instance, lymphoid-deficient cells (α-HSCs) are present at very low frequencies (<5% of all HSCs) in fetal liver, and their levels increase gradually with age, so that just before birth they constitute around 15% of all HSCs in fetal bone marrow. After birth, their levels correspond to 20% of HSCs, and in young adults their levels reach 25–30% of the HSC pool. In old mice, α-HSCs correspond to 45% of all HSCs ^25^. The presence of α, β, γ, and δ HSCs in humans is less clear.

## How are HSCs regulated?

HSC viability, self-renewal, proliferation, commitment, and differentiation depend on both intrinsic and extrinsic elements. The former include a variety of regulatory molecules present within the cell, whereas the latter comprise different cell types and their products, which create the microenvironment in which HSCs grow. Thus, we can say that the function of HSCs is controlled by intrinsic cell regulators, which, in turn, are modulated by external signals ^26^. Among the intrinsic regulators of stem cell function, we find nuclear transcription factors that control gene expression (for instance, the transcription factor SCL is essential for HSC survival, self-renewal, and quiescence ^27^); molecular regulators of the cell cycle, including some cyclins and cyclin-dependent kinases ^28^ (for instance, CDK6 is absent in long-term HSCs, which keeps them quiescent even in the presence of mitogenic stimulation; in contrast, short-term HSCs express high levels of CDK6, and this results in rapid entry into the cell cycle upon mitogenic stimulation ^29^); the proteins responsible for setting up symmetric and asymmetric cell divisions, such as Musashi-2 ^30^; molecules that act as mitotic clocks that set up the number of rounds of division (HSCs express high levels of telomerase, thus the length of their telomeres does not decrease as rapidly as in more mature cells ^31^); and epigenetic regulators controlling the structure and organization of DNA and chromatin ^32^.

In postnatal life, blood cell formation takes place primarily in the bone marrow. Here, stem cells are surrounded by different cell types, including stromal (e.g., mesenchymal stromal cells [MSCs], osteoblasts, fibroblasts, adipocytes, macrophages, and endothelial cells) and accessory (e.g., lymphocytes) cells. All of these different cell types form a unique environment, known as the HSC niche, that is responsible for providing HSCs with the right conditions for their growth ^33, 34^. Interestingly, recent evidence indicates that there are, in fact, several hematopoietic niches within the marrow microenvironment, including endosteal, vascular, and perivascular niches, which exert differential effects on HSCs ^35^. The composition of each one of these niches is different, e.g., the endosteal niche consists mainly of osteoblasts, whereas the vascular niche consists of endothelial cells. The perivascular niche, in turn, contains both MSCs and Cxcl12-abundant reticular (CAR) cells ^35^. Current evidence indicates that most of the HSCs residing in the bone marrow (around 85% of marrow HSCs) are located within 10 µm of a sinusoidal vessel ^36^ and that cell fate is dictated mainly by elements of the perivascular niche ^35^.

The cells that form part of the stem cell niche are able to produce and secrete a wide array of proteins – including extracellular matrix, cytokines, and chemokines – that influence stem cell behavior ^37^. Cytokines exert their effects via specific molecules (receptors) located on the cell surface ^38^, and they can be presented to their target cells as soluble or as membrane-bound molecules. The fact that some cytokines are presented as membrane-bound proteins implies that direct cell-to-cell interactions must take place between the cytokine-producing cell and the cytokine receptor-bearing cell. It has been suggested that the primary action of cytokines on stem cells is to prevent cell death and to promote cell division ^38^.

## Controlling HSC fate

In stem cell biology, the balance between self-renewal and differentiation is of key relevance. Cell fate decisions are associated with changes in gene expression and are controlled by the action of transcription factors ^39^. Gene expression changes are usually accompanied (specifically preceded) by epigenetic changes in regulatory regions ^40^. It is noteworthy, however, that the initial changes usually occur without *de novo* transcription and are mediated by the asymmetric distribution of cell fate determinants ^41^.

In humans, several transcription factors have been associated with the HSC state, including ID genes, SOX8, SOX18, and NFIB. In contrast, factors such as MYC and IKZF1 have been implicated in differentiation into MPPs ^21^. HOXB4 has also been found to be important in HSC biology, both in mice and in humans. Indeed, overexpression of such a factor in mouse HSCs induces symmetric divisions, which result in a 1000-fold expansion in HSC numbers ^42^. BMI1, a polycomb-group factor, increases the multilineage potential of human HSCs, as well as their replating capacity; in contrast, *bmi1* deletion results in loss of clonal potential ^43, 44^. Other genes whose expression favors self-renewal and confers increased repopulation potential include *hes1* and *hlf* ^45^, as well as *notch* ^46^. Activation of certain genes and pathways has been implicated in the loss of HSC potential. For instance, activation of the mTOR pathway results in the loss of HSC self-renewal ^47^; similarly, BATF activation decreases self-renewal capacity and induces lymphoid differentiation ^48^.

Today, we know that HSC fate choices are greatly influenced and controlled by epigenetic changes. For instance, an increase in H4K16Ac levels results in inhibition of Cdc42, and this, in turn, results in restoration of the B cell lineage output in aged HSCs ^49^. Increased levels of H3K9me2 mark the onset of HSC lineage commitment, whereas inhibition of G9a improves HSC maintenance ^50^. Non-coding RNAs are also key regulators of HSC biology. MiRNA-22, for example, is a powerful inducer of HSC maintenance and self-renewal ^51^, and very recently miRNA-126 was shown to play a key role in the self-renewal capacity and outcome of HSCs ^52^.

## Implications and challenges

Understanding the elements and mechanisms that regulate HSC populations is of significant relevance at two different levels. On the one hand, it is important in our knowledge regarding blood cell production under both normal and pathological states; thus, it helps us decipher the steps and pathways that lead to disorders such as myelodysplasia or leukemia. Indeed, particular mutations in several transcription factors have been implicated in the pathophysiology of such disorders (reviewed in 4). On the other hand, it is important in the development of therapeutic strategies. For instance, knowledge of the Notch pathway has led to the development of laboratory strategies for the *ex vivo* expansion of HSCs and HPCs from human cord blood ^53, 54^.

Research on the regulation of HSC populations still has to face several challenges. One of the most obvious is related to the development of therapeutic strategies using specific regulatory molecules and intracellular pathways as targets; for example, WNT or Notch pathways. Another one would be trying to understand aging of the HSC pool at the single cell level. In this regard, it will be of the most importance in the development of single cell RNA technologies, some of which have already been worked out, to fully understand the processes of gene regulation in HSCs from young and old individuals. Promising and exciting years are yet to come.
